# Advanced Strategies for End-Stage Heart Failure: Combining Regenerative Approaches with LVAD, a New Horizon?

**DOI:** 10.3389/fsurg.2015.00010

**Published:** 2015-04-07

**Authors:** Cheyenne C. S. Tseng, Faiz Z. Ramjankhan, Nicolaas de Jonge, Steven A. J. Chamuleau

**Affiliations:** ^1^Department of Cardiology, Division Heart and Lungs, University Medical Center, Utrecht, Netherlands; ^2^Interuniversity Cardiology Institute of the Netherlands, Utrecht, Netherlands; ^3^Department of Cardio-thoracic Surgery, Division Heart and Lungs, University Medical Center, Utrecht, Netherlands

**Keywords:** heart failure, ventricular-assist device, mechanical circulatory support, regenerative therapies, cell therapy, cardiac recovery

## Abstract

Despite the improved treatment of cardiovascular diseases, the population with end-stage heart failure (HF) is progressively growing. The scarcity of the gold standard therapy, heart transplantation, demands novel therapeutic approaches. For patients awaiting transplantation, ventricular-assist devices have been of great benefit on survival. To allow explantation of the assist device and obviate heart transplantation, sufficient and durable myocardial recovery is necessary. However, explant rates so far are low. Combining mechanical circulatory support with regenerative therapies such as cell (-based) therapy and biomaterials might give rise to improved long-term results. Although synergistic effects are suggested with mechanical support and stem cell therapy, evidence in both preclinical and clinical setting is lacking. This review focuses on advanced and innovative strategies for the treatment of end-stage HF and furthermore appraises clinical experience with combined strategies.

## Introduction

**Figure 1:**
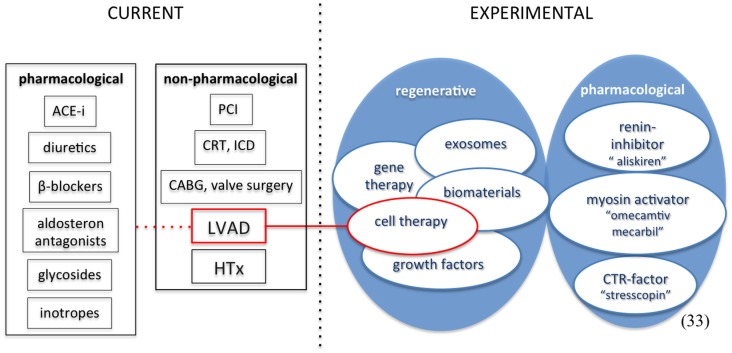
**Current and experimental heart failure therapy**. ACE-i, angiotensin-converting-enzyme inhibitor; PCI, percutaneous coronary intervention; CRT, cardiac resynchronization therapy; ICD, implantable cardioverter defibrillator; CABG, coronary artery bypass graft; HTx, heart transplantation; CTR-factor, cortico-trophin-releasing factor.

Heart failure (HF) is a progressive disease with an important economic burden on today’s healthcare. After initial injury, progressive worsening maladaptive (cellular and structural) changes result in a process called ventricular remodeling, eventually leading to diminished cardiac function (1, 2). According to the Framingham study, the incidence of HF has remained stable since the 1970s (3). Despite this unchanged incidence, the population of HF patients is growing, affecting up to around 23 million patients worldwide, due to various aspects. Improvement in the acute therapy of myocardial infarction (MI) has played a major role in survival rates. Other non-pharmacological treatment options such as ICD therapy have further decreased mortality. In addition, the widespread use of ACE-inhibitors, ATII-blockers, beta-blockers, and aldosterone-antagonists, but also cardiac resynchronization therapy further enhanced survival among HF patients. These developments in combination with an aging population translate into an increase in the prevalence of chronic “end-stage HF” (4, 5). Although not clearly defined, according to the guidelines for heart transplantation, heart transplantation should be considered in patients with severe symptoms of HF, intractable angina, or rhythm disturbances, without any alternative form of treatment available and with a poor prognosis (6). Concerning the guidelines for HF, there are different types of management approaches, which can be broadly subdivided in three groups, (1) general/non-pharmacological measures, (2) pharmacological therapy, and (3) devices and surgery (7, 8). The only current available therapy for end-stage HF is heart transplantation. Opposed to an increasing demand for donor hearts, the number of heart transplantations in Europe has diminished in recent years. In the Netherlands especially, decreasing mortality after traffic accidents, older donors, and shift from heart-beating donation to non-heart-beating procedures gave rise to a further decreasing amount of donors (6). To compensate for the shortcoming of donors, novel therapeutic strategies are inevitable. Experimental regenerative therapies, intended to restore functional cardiac cells and myocardial function are of great interest (9, 10). An overview of heart failure treatment is depicted in Figure 1. For some patients, mechanical circulatory support (MCS) with a ventricular-assist device (VAD) is an option. This review will focus on current and novel, advanced therapeutic strategies for end-stage HF.

## Current Therapies for End-Stage Heart Failure

### Heart transplantation

In European countries that are represented by the European Society of Cardiology (ESC), there are estimated to be over 10 million patients with HF (7). For the Netherlands, this number is believed to be between 100.000 and 150.000 patients, and is expected to rise to approximately 195.000 in 2025 (11). These numbers are probably underestimated and lack accuracy due to the absence of a uniform definition for HF. Easier to determine is the number of patients waiting for a donor heart. Eurotransplant is the international collaborative framework responsible for allocation of donor organs in the Netherlands, Austria, Belgium, Croatia, Germany, Hungary, Luxembourg, and Slovenia. Annual statistics show a rising number of patients on the waiting list, with an actual number of 1250 patients at the end of December 2013, a 2.5-fold increase compared to 2000 (12). With a total of 563 heart transplantations in 2013, the scarcity of donor hearts is evident. In the Netherlands, the same trend is seen. Added up with the progressive decline in the amount of donors, heart transplantation will not relieve the burden of HF on healthcare.

### Mechanical support

As briefly stated in the introduction, MCS with a VAD is a possibility for some patients. VADs can be used as a bridge to transplantation, recovery or decision, and as destination therapy. These mechanical pumps partially or completely take over ventricular function to support circulation. Either the left ventricle (LV), right ventricle (RV), or both ventricles can be unloaded. Predominantly, left ventricular-assist devices (LVADs) are implanted because of disappointing results of biventricular-assist device (BIVAD) support (13). Since the first successful implantation of a VAD in 1966 by DeBakey (14), mechanical support has shown to be of great value in survival of patients with advanced HF. The landmark REMATCH trial (15) compared the long-term use of the first generation, pulsatile LVADs with optimal medical therapy in end-stage HF and showed significantly improved survival with an absolute reduction in mortality rate of 27% at 1 year (16). Two major factors causing a low 2-year survival rate of 23% in the LVAD group were infection and mechanical device-failure (16). Since 2006, continuous-flow assist devices are implanted, with much better results (17). Lahpor et al. (18) explored the outcomes of more than 400 patients with this second generation, continuous-flow device and found no mechanical failure, a low incidence of neurological complications but still major infectious and bleeding complications. Although the mean duration of support was significantly higher due to the shortage of donor hearts, overall survival is comparable to other studies (17, 18). Permanent mechanical support, or LVAD as destination therapy, is an option for patients with contraindications for heart transplantation, but reimbursement differs per country (19). In addition to the financial aspects, durable LVAD support as a therapy for end-stage HF is still hampered by substantial bleeding (2.69 events/pt-year) and thromboembolic events (0.31 events/pt-year), as well as inflammatory complications (2.34 events/pt-year) (16, 18, 20–22). The fifth Interagency Registry for Mechanically Assisted Circulatory Support (INTERMACS) analysis demonstrated a sixfold increase of pump exchanges for pump thrombosis between 2011 and 2012, clinically concerning due to the associated higher mortality rates (23, 24). These findings emphasize the importance of restricting long-term support only for those who really need that and stimulating myocardial recovery and device explantation in as many patients as possible. While initially ventricular remodeling in end-stage HF was held to be irreversible, multiple analyses have shown high percentages of “reverse remodeling” but only low numbers of myocardial recovery (1, 2, 25). Although the influence on end-organ perfusion and unloading is similar with pulsatile versus continuous-flow support, conflicting literature exists regarding their influence on recovery (26). In the current clinical setting, LVADs infrequently lead to sufficient myocardial recovery to allow device explantation, i.e., function as a bridge to recovery (BTR). In a retrospective review with patients receiving MCS as a bridge to transplantation, recovery occurred in less than 5% of patients (27). An explantation rate of 9% was described by the LVAD working group, mostly in younger patients with recent onset HF of non-ischemic origin (2). These results correspond to prior data showing higher percentages of myocardial recovery in patients with non-ischemic cardiomyopathy (28). Yacoub et al. aimed at the process of “physiological hypertrophy” and “reverse remodeling” to maximize the rate of cardiac recovery by using LVAD support in combination with a specific sequence of pharmacological therapy, including beta-2-agonist Clenbuterol (29). A small cohort of 15 patients receiving MCS for non-ischemic cardiomyopathy without acute myocarditis, were treated with the particular sequence of medication that resulted in sufficient recovery to meet explantation criteria in 11 patients (73%). In most cases, improvement maintained for more than 4 years (30). Long-term outcomes of patients bridged to recovery versus bridged to transplantation were investigated (31) to review the results of an aggressive attempt at stimulating myocardial recovery. Particularly patients with non-ischemic cardiomyopathy profit from aggressively inducing reversal of HF. The rate of device explantation was 20.5%, much higher than other data so far (2, 31, 32).

## Experimental Regenerative Therapies

A concise concept of different regenerative approaches, present experience, and associated hurdles for clinical application will be discussed. Experimental pharmacological therapies (33) are beyond the scope of this review.

### Cell therapy

Various cell populations and delivery strategies have been examined for their cardiac repair and regenerative capacity in the last decades. Stem cells can be derived from blood, bone marrow, skeletal muscle, adipose tissue, embryonic sources, or cardiac tissue (34, 35). Initially, stem cells were presumed to replace damaged cardiomyocytes. Instead, currently, the major mechanism of action is assumed to be trough paracrine factors leading to decreased neurohormonal activation and apoptosis, better Ca2^+^-handling, stimulation of neovascularization, and activation of endogenous cardiac-resident cells (36–38). Most clinical experience with cell therapy is gained in ischemic heart disease with unselected bone marrow-derived mononuclear cells (BMMNCs). Results in clinical setting are modest but significant with greatest improvements in the lowest LV ejection fraction (EF) at baseline (34, 38–45). The discordance with preclinical data is not fully explained, but poor cell retention and survival plus possible malfunctioning of bone marrow-derived cells in patients with HF are alleged to play a role in these somewhat disappointing findings (34, 46–48). The discovery of so-called endogenous cardiac stem cells (CSCs) (49) and evidence that cardiomyocytes have renewal capacity (49–51) have provided a new therapeutic approach, to stimulate the endogenous CSCs since these cells are programed to reconstitute cardiac tissue. Encouraging results were shown in the SCIPIO trial (autologous CSCs) (52) and in the CADUCEUS trial (autologous cardiosphere-derived cells) (53). Compared to ischemic heart disease, limited clinical data is available regarding efficacy of cell therapy in dilated cardiomyopathy. Results in this population seem mostly positive, though heterogeneity of the population, procedures, and outcome parameters prohibit extrapolation (54). A novel cell-based technology in which somatic cells (all cells in the body except germ cells) are modified or reprogramed into a special type of stem cell, called induced pluripotent stem cell (iPS), is in development (35, 55). This technique is already applied for other purposes but is fairly unknown as therapeutic. To improve clinical success of cell therapy, better understanding of the primary mechanism and best cell type are fundamental (10, 36, 38, 41). In addition, knowledge about optimal timing, dosing, and delivery strategies, including better cell retention and survival, is essential (35, 46, 56).

### Growth factors

Growth hormones (GHs) are essential for normal myocardial and endothelium development, and for the maintenance of function (57, 58). Endogenous CSCs can be activated by growth factors in the infarcted heart as shown in rodents (59) and in a porcine model (60) of acute MI (34, 59–61). Vascular endothelial growth factor (VEGF) and granulocyte–macrophage colony-stimulating factor (GM-CSF) augment levels of endothelial progenitor cells (EPCs) and improve neovascularization (34). Hepatocyt growth factor (HGF) promotes cell migration, insulin-like growth factor-1 (IGF-1) is mitogenic and antiapoptotic, stimulates myocyte formation, and reduces myocyte death after infarction (59). GH therapy in chronic setting seems rational since part of the neurohormonal disturbances in HF lies in the GH/IGF-1 signaling axis (58). Whereas animal studies demonstrate beneficial effects of growth factor therapy (34, 62), clinical data about the efficacy of growth factors in HF patients is conflicting (58). In a small group of patients (*n* = 13) with severe coronary artery disease and refractory angina, treatment with high doses of VEGF temporarily enhanced myocardial perfusion (63). A preliminary study by Fazio et al. (64) showed improved cardiac output in seven patients with dilated cardiomyopathy treated with GH, whilst other studies examining the effects of exogenous GH in HF yielded no beneficial effects on cardiac function (65–68). A more recent clinical trial (69) with granulocyte-colony-stimulating factor after MI, although appearing to improve LV function, was terminated because of high incidence of in-stent restenosis in this treated group. A safety and efficacy trial with IGF-1 is currently recruiting patients with acute MI (Clinical trial info: NCT 01438086).

### Gene therapy

Interest for experimental gene therapy in cardiovascular disease has grown in the last 10 years. The most relevant systems targeted to restore function of failing cardiomyocytes are (1) the B-adrenergic system, (2) Ca^2+^ cycling proteins, (3) homing stem cells, and (4) cell death (70). The first clinical, phase 2A safety study (CUPID), with adeno-associated virus (AAV) type 1/sarcoplasmic reticulum Ca^2+^-ATPase (SERCA2a) suggests a positive effect on LV function (71), but the therapeutic potential will become apparent as a phase 2b trial is ongoing (clinicaltrials.gov: NCT 01643330) (72). Other targets that have been taken forward toward clinical trials include adenylyl cyclase type 6 (clinicaltrials.gov: NCT 00787059) and stromal cell-derived factor-1 (SDF-1) (clinicaltrials.gov: NCT 01082094) (70). As more molecular targets associated with HF are discovered, more effective gene therapy is expected to emerge (70). Another concept in gene therapy for HF concerns microRNA (miRNA). These are small non-coding RNAs that bind to specific target mRNAs, thereby suppressing protein expression (73, 74). The capacity to manipulate miRNA expression and function, together with the fact that their function is heightened under pathophysiological conditions, make them attractive candidates for therapeutic manipulation. Either inhibitors (antimiR) or mimics of miRNA are of interest (73). Several small and large animal studies have targeted relevant miRNA (-families) (73). For example, miRNA-208a (cardiac remodeling), miRNA-21 (cardiac hypertrophy and fibrosis), miRNA-15 (cardiomyocyte apoptosis and regeneration), and miRNA-92a (angiogenesis and regeneration) have been found to play a role in cardiovascular pathology (74–77). Hinkel et al. demonstrated improved recovery after ischemia/reperfusion injury by inhibiting miR-92a by LNA-based miRNA inhibitor in a pig model (74). Challenges in miRNA therapy essentially concern the pleiotropy and multiplicity of miRNA that needs intensive research, since only target tissue is examined in all studies. Next to that, feasibility of adequate dosing has to be assessed (70).

### Exosomes

These small membrane vesicles (40–100 nm), endosomal-derived and extracellularly released by many cells, are involved in intercellular communication (78, 79). Although discovered 30 years ago (80), major interest in exosomes and their function in regenerative medicine recently emerged. In response to injury, extracellular microvesicles are released from activated platelets and apoptotic endothelial cells, suggesting not only therapeutic but also diagnostic value (81). Special attention for exosomes derived from cardiac progenitor cells has originated from the postulated paracrine effects of cell-based therapy, mainly regarding the release of growth factors, cytokines, and chemokines (78, 81). Exosomes derived from cardiomyocyte progenitor cells are proposed to play a role in cardiac protection (78). In mice as well as in a porcine model of ischemia/reperfusion injury, mesenchymal stromal cell-derived exosomes reduced myocardial damage (78, 82). While acknowledged to target via transfer of proteins or genetic materials, the role of exosomes in cardiac injury is far from clear (79). Further research on the production and content sorting of exosomes and their effect on target (and non-target) cells is crucial (79, 81).

### Biomaterials

Another recent topic in regenerative therapy for cardiovascular disease is the use of biomaterials. Multiple scaffolds, naturally derived and synthetic, are used. Therapeutic ability is suggested for MI, prevention of remodeling, and in consequence prevention of ischemic HF in small and large animal models (83–85). Whilst originally tissue-engineered cardiac patches were of interest, research in the area of injectable biomaterials is rapidly evolving (47, 61, 83, 86, 87). The prospective profit of biomaterials is two-sided, either to stimulate endogenous repair and regeneration or to provide a vehicle to support delivery of other therapeutics (e.g., cells, growth factors), generating greater cell retention and survival (47, 86). Gelatin microspheres have been shown to be a feasible carrier for cardiomyocyte progenitor cells and growth factors, resulting in improved engraftment and cell survival in mice (Feyen. Thesis: Strategies to improve cardiac cell therapy. Chapter 8: Gelatin microspheres as carriers for cardiac progenitor cell and growth factor to the ischemic myocardium, unpublished, 2014). Dai et al. studied the effect of non-cellular hydrogels versus cell therapy in a rat model of chronic ischemia and showed similar increases in EF and thereby potential of hydrogels alone (84). No clinical trials have yet been performed with biomaterials. The challenge of this therapy is the delivery, mainly relating to the solubility during the procedure while the hydrogel has to become gel-like after injection in the myocardium.

## Combined Mechanical Support and Regenerative Therapies

Following unloading of the ventricle, a complex network of changes on molecular, cellular, tissue, and organ level arises (32, 88–94). Although the exact mechanism of reversal of HF during LVAD support is unclear, the effect of ventricular volume and pressure unloading together with improved neurohormonal and cytokine activation are thought to induce reverse remodeling (1, 92, 95). In a study comparing isolated human myocytes of failing hearts with and without prior LVAD showed increased contractile properties and beta-adrenergic responsiveness after LVAD support (96). Immunohistochemical analysis of the contractile myofilaments after LVAD implantation uncovered improved staining pattern of all thin contractile proteins and titin, however structural myocyte damage was persisting (89). Significant improvement of the proliferation/apoptosis balance by ventricular unloading has been shown in a mouse model of ischemic HF (95). Also, specific changes in gene expression of cytoskeletal proteins after LVAD support have been seen in recovered versus non-recovered myocardium (91). The beneficial effect on LV function appears to deteriorate over time (2), suggesting that combining mechanical support with other, regenerative, therapeutic strategies like GHs, gene therapy, or cell therapy might hold the key to better long-term results (92). The unloaded ventricle provides a less hostile milieu and thereby a potentially more appropriate platform for different regenerative therapies. Along the same lines, the combined approach of biventricular pacing and BMMNCs in ischemic HF indicated a significant and clinically relevant improvement in cardiac function in comparison with BMMNCs alone, while CRT showed no impact on perfusion (97). The rationale for this approach is that electrical stimulation might promote cell differentiation.

### Preclinical experience

Up to date, a representative large animal model of chronic HF with myocardial unloading is lacking. The majority of LVAD studies was performed in healthy animals with only a few studies in chronically failing models (98). Preclinical experience consists of several (b)ovine ischemic HF models, induced by either coronary microembolization, coronary ligation, or ameroid constriction (99–103). Non-ischemic HF models include a pressure and volume overload model caused by aortic constriction, respectively, mitral regurgitation via chordae rupture (98, 104). Last-mentioned models have the disadvantage of required thoracotomy, undesirable in case of future device implantation. Other methods like pacing, pharmacotherapeutic induced (doxorubicin), direct shock, and cardiotoxins are not reflective of human HF (98). Recreating a model similar to human etiology remains a challenge. To advance innovative and clinically applicable strategies for cardiac regeneration, suitable preclinical research is inevitable. Not only to test combined unloading and regenerative therapies, but also to direct future mechanical support and treatment of earlier stage HF. Thereafter, different regenerative therapies must be evaluated in such a model.

### Clinical experience

Combined mechanical unloading and regenerative therapy in clinical setting has only been examined with cell therapy. The results of these studies were systematically reviewed as shown in Table 1 (39, 40, 105–111). A total of 50 patients have been treated with the combinational strategy. The limited data illustrate that in all cases that LVAD was explanted, an extracorporeal device was used. Usually, percutaneous support is initiated when myocardial recovery is expected. However, Sawa et al. (105) describe a case where a patient with idiopathic dilated cardiomyopathy did not show enough improvement in LVEF for explantation 7 months after starting MCS. After additional cell transplantation, LV improved to a reasonable function that sustained for at least 1.5 years. All studies, except Ascheim et al., used autologous cells, either bone marrow-derived or skeletal myoblasts, mainly in patients with ischemic cardiomyopathy. The first and only randomized trial with allogeneic mesenchymal precursor cells in ischemic and non-ischemic HF shows encouraging results when it comes to efficacy, but safety regarding sensitization is concerning, especially when the aim is to increase the amount of cells in future studies (111). No results of the combination of SERCA gene therapy and MCS have yet been reported (clinicaltrials.gov: NCT 000534703). Accordingly, the combination of MCS and cell therapy is promising as both therapies share action mechanisms and might possess synergistic effects (39, 40, 92, 105, 107–111). Focusing on this combination provides not only a point of reference to gain more success in bridging to recovery but also the unique opportunity to analyze the myocardium in case of heart transplantation, which can broaden understanding in the process of ventricular reverse remodeling and myocardial recovery. In patients awaiting heart transplantation, allogeneic cell therapy should only be considered with great precaution because of immunologic sensitization (111).

**Table 1:** **Clinical experience of LVAD combined with cell therapy**.

Study type (Reference)	*n*	Etiology CMP	Cell type (and timing)	Clinical outcome	Measured effect
Phase I (111)	20	Ischemic and non-ischemic	Allogeneic MPCs (concomitant)	Increased weaning frequency and duration	Safe/efficacy
Case report (105)	1	Dilated	Autologous skeletal myoblasts (+16 months)	LVAD explantation	LVEF increased
Phase I (110)	4	Ischemic	Autologous skeletal myoblasts (concomitant)	1 LVAD explantation, 3 non-cardiac deaths	*n* = 2 LVEF increased
Case series (108)	2	Ischemic	Autologous BMMNCs (concomitant)	1 Improved perfusion, 1 unknown	Perfusion improved
Case report (107)	1	Ischemic	Autologous skeletal myoblasts (+3 months)	Death + 466 days (sepsis)	Increased EF
Case series (109)	10	Ischemic	Autologous BMMNCs (concomitant)	1 LVAD explantation, 3 HTx, 2 deaths	*n* = 1 increased EF
Case report (106)	1	Ischemic	Autologous BMMNCs (+99 days)	LVAD explantation	Increased EF and perfusion
Phase I (40)	6	Ischemic	Autologous skeletal myoblasts (concomitant)	4 HTx, 3 deaths	Safe/feasible
Phase I (39)	5	Ischemic	Autologous skeletal myoblasts (concomitant)	3 HTx, 1 DT, 1 death	Safe/feasible

*CMP, cardiomyopathy; MPCs, mesenchymal progenitor cells; BMMNCs, bone marrow-derived mononuclear cells; HTx, heart transplantation; DT, destination therapy*.

*Systematic search LVAD/SCT: search detail: “heart-assist devices” [MeSH Terms] OR (“heart-assist devices” [MeSH Terms] OR (“heart-assist” [All Fields] AND “devices” [All Fields]) OR “heart-assist devices” [All Fields] OR (“heart” [All Fields] AND “assist” [All Fields] AND “device”[All Fields]) OR “heart-assist device” [All Fields]) AND (“cell- and tissue-based therapy” [MeSH Terms] OR (“cell-” [All Fields] AND “tissue-based” [All Fields] AND “therapy” [All Fields]) OR “cell- and tissue-based therapy” [All Fields] OR (“cell” [All Fields] AND “therapy” [All Fields]) OR “cell therapy” [All Fields])*.

*In total: 195 hits, excluding review/animal/Japanese/no combination → 9 included articles*.

## Future Perspective

The rapidly developing field of regenerative therapies enables various combinations with LVAD support (e.g., hydrogel loaded with exosomes or growth factors combined with microspheres). Considering the different etiologies of HF, the most pronounced effect of combined cell therapy, biomaterials, and mechanical unloading could be expected in patients with ischemic HF. The rationale is that the ischemic myocardium will benefit most from the paracrine effects leading to angiogenesis. The combination with biomaterials might positively enlarge efficacy by higher retention rates, and perhaps through a direct therapeutic effect of the biomaterial. Gene therapy in combination with (biomaterials and) MCS is more probable to enhance myocardial function of patients with dilated cardiomyopathy. The advancements in assist devices will help to uncover the most optimal technology to stimulate recovery and reduce adverse events. Cheng et al. suggest that pulsatile flow support might have better results with regard to recovery, due to the less affected vascular reactivity in the presence of a pulse pressure (26). The absence of arterial pulsatility leads to stiff unresponsive arteries (102). Moreover, the development of algorithms for continuous-flow-LVADs to generate a pulse pressure is very intriguing, also for the possible influence on adverse events (26). The increasing rate of permanent LVAD support will lead to more clinical data regarding recovery rates and adverse events. However, the small number of patients included in LVAD trials and the lack of an illustrative preclinical model, makes moving forward to clinical application time-consuming. Besides testing of combined therapeutic strategies, preclinical research is also inevitable to gain more understanding of the types of support in the setting of myocardial recovery.

## Conclusion

Since heart transplantation, the gold standard therapy for end-stage HF, is not sufficiently available, other advanced therapeutic approaches are crucial. LVADs provide a bridge for patients awaiting heart transplantation or myocardial recovery. Rates of successful and durable recovery are very low, but this can be stimulated pharmacologically. Better-sustained results could be expected from combining LVADs with regenerative therapies such as gene therapy, biomaterials, and cell-based therapies. Especially, cell therapy for the treatment of heart disease has been extensively studied, showing promising results. The small number of LVAD patients does not allow clinical testing of the numerous potential combinations of therapies. A clinically relevant animal model of unloading should be established for preclinical testing of these regenerative approaches. Regarding current experience in the reversal of HF with combined LVAD and cell therapy, future clinical research should focus on placebo-controlled studies in patients undergoing LVAD implantation.

## Conflict of Interest Statement

The authors declare that the research was conducted in the absence of any commercial or financial relationships that could be construed as a potential conflict of interest.

## Abbreviations

BIVAD, biventricular-assist device; BMMNCs, bone marrow-derived mononuclear cells; CSCs, cardiac stem cells; EF, ejection fraction; ESC, European Society of Cardiology; GHs, growth hormones; HF, heart failure; HGF, hepatocyt growth factor; IGF-1, insulin-like growth factor-1; INTERMACS, Interagency Registry for Mechanically Assisted Circulatory Support; LV, left ventricle or ventricular; LVAD, left ventricular-assist device; MCS, mechanical circulatory support; MI, myocardial infarction; miRNA, microRNA; VAD, ventricular-assist device.

## References

[1] Levin H Oz M Chen J Packer M Rose EA Burkhoff D. Reversal of chronic ventricular dilation in patients with end-stage cardiomyopathy by prolonged mechanical unloading. Circulation (1995) 91(11):2717–20. 10.1161/01.CIR.91.11.2717 7758175

[2] Maybaum S Mancini D Xydas S Starling RC Aaronson K Pagani FD Cardiac improvement during mechanical circulatory support: a prospective multicenter study of the LVAD working group. Circulation (2007) 115(19):2497–505. 10.1161/CIRCULATIONAHA.106.633180 17485581

[3] Redfield M Heart failure – an epidemic of uncertain proportions. N Engl J Med (2002) 347(18):1442–4 10.1056/NEJMe020115 12409548

[4] Levy D Kenchaiah S Larson MG Benjamin EJ Kupka MJ Ho KKL Long-term trends in the incidence of and survival with heart failure. N Engl J Med (2002) 347(18):1397–402. 10.1056/NEJMoa020265 12409541

[5] De Jonge N Vantrimpont PJ Heart failure: chapter 8. Treatment of end-stage heart failure. Neth Heart J (2004) 12(12):548–54. 25696289 PMC2497214

[6] De Jonge N Kirkels JH Klöpping C Lahpor JR Caliskan K Maat AP Guidelines for heart transplantation. Neth Heart J (2008) 16(3):79–87 10.1007/BF03086123 18345330 PMC2266869

[7] Remme WJ Swedberg K Guidelines for the diagnosis and treatment of chronic heart failure. Eur Heart J (2001) 22(17):1527–60 10.1053/euhj.2001.2783 11492984

[8] Remme WJ Swedberg K Comprehensive guidelines for the diagnosis and treatment of chronic heart failure. Task force for the diagnosis and treatment of chronic heart failure of the European Society of Cardiology. Eur J Heart Fail (2002) 4(1):11–22 10.1016/S1388-9842(01)00231-8 11812661

[9] Mason C Dunnill P A brief definition of regenerative medicine. Regen Med (2008) 3:1–5 10.2217/17460751.3.1.1 18154457

[10] Du Pré BC Doevendans PA van Laake LW. Stem cells for cardiac repair: an introduction. J Geriatr Cardiol (2013) 10(2):186–97. 10.3969/j.issn.1671-5411.2013.02.003 23888179 PMC3708059

[11] Koopman C Van Dis I Bots M Vaartjes I Feiten en cijfers Hartfalen. *Nederlandse Hartstichting* (2012). Available from: https://www.hartstichting.nl/downloads/factsheet-hartfalen

[12] Eurotransplant International Foundation. Annual Report 2013. Rahmel A, editor. Leiden: Eurotransplant Foundation (2013).

[13] Cleveland JC Naftel DC Reece TB Murray M Antaki J Pagani FD Survival after biventricular assist device implantation: an analysis of the interagency registry for mechanically assisted circulatory support database. J Heart Lung Transplant (2011) 30(8):863–9 10.1016/j.healun.2011.04.004 21621423

[14] Liotta D Early clinical application of assisted circulation. Tex Heart Inst J (2002) 29(3):229–30. 12224734 PMC124772

[15] Rose EA Moskowitz AJ Packer M Sollano JA Williams DL Tierney AR The REMATCH trial: rationale, design, and end points. Ann Thorac Surg (1999) 67(3):723–30. 10.1016/S0003-4975(99)00042-9 10215217

[16] Rose EA Gelijns AC Moskowitz AJ Heitjan DF Stevenson LW Dembitsky W Long-term use of a left ventricular assist device for end-stage heart failure. N Engl J Med (2001) 345(20):1435–43. 10.1056/NEJMoa012175 11794191

[17] Slaughter MS Rogers JG Milano CA Russell SD Conte JV Feldman D Advanced heart failure treated with continuous-flow left ventricular assist device. N Engl J Med (2009) 361(23):2241–51. 10.1056/NEJMoa0909938 19920051

[18] Lahpor J Khaghani A Hetzer R Pavie A Friedrich I Sander K European results with a continuous-flow ventricular assist device for advanced heart-failure patients. Eur J Cardiothorac Surg (2010) 37(2):357–61. 10.1016/j.ejcts.2009.05.043 19616963

[19] Neyt M Van den Bruel A Smit Y De Jonge N Erasmus M Van Dijk D Cost-effectiveness of continuous-flow left ventricular assist devices. Int J Technol Assess Health Care (2013) 29(3):254–60. 10.1017/S0266462313000238 23763844

[20] Stulak JM Lee D Haft JW Romano MA Cowger JA Park SJ Gastrointestinal bleeding and subsequent risk of thromboembolic events during support with a left ventricular assist device. J Heart Lung Transplant (2014) 33(1):60–4. 10.1016/j.healun.2013.07.020 24021944

[21] Kirklin JK Naftel DC Cantor RS Myers SL Clark ML Collum SC Quarterly Statistical Report 2014 3rd Quarter. INTERMACS Interagency Registry for Mechanically Assisted Circulatory Support. Birmingham: The Data Collection and Analysis Center University of Alabama (2014).

[22] Kirklin JK Naftel DC Pagani FD Kormos RL Stevenson LW Blume ED Sixth INTERMACS annual report: a 10,000-patient database. J Heart Lung Transplant (2014) 33(6):555–64. 10.1016/j.healun.2014.04.010 24856259

[23] Kirklin JK Naftel DC Kormos RL Pagani FD Myers SL Stevenson LW Interagency registry for mechanically assisted circulatory support (INTERMACS) analysis of pump thrombosis in the HeartMate II left ventricular assist device. J Heart Lung Transplant (2014) 33(1):12–22. 10.1016/j.healun.2013.11.001 24418730

[24] Mehra MR Stewart GC Uber PA Pharm D. The vexing problem of thrombosis in long-term mechanical circulatory support. J Heart Lung Transplant (2014) 33(1):1–11. 10.1016/j.healun.2013.12.002 24418729

[25] Maybaum S Kamalakannan G Murthy S. Cardiac recovery during mechanical assist device support. Semin Thorac Cardiovasc Surg (2008) 20(3):234–46. 10.1053/j.semtcvs.2008.08.003 19038734

[26] Cheng A Williamitis CA Slaughter MS. Comparison of continuous-flow and pulsatile-flow left ventricular assist devices: is there an advantage to pulsatility? Ann Cardiothorac Surg (2014) 3(6):573–81. 10.3978/j.issn.2225-319X.2014.08.24 25512897 PMC4250555

[27] Mancini DM Beniaminovitz A Levin H Catanese K Flannery M DiTullio M Low incidence of myocardial recovery after left ventricular assist device implantation in patients with chronic heart failure. Circulation (1998) 98(22):2383–9. 10.1161/01.CIR.98.22.2383 9832482

[28] Simon MA Kormos RL Murali S Nair P Heffernan M Gorcsan J Myocardial recovery using ventricular assist devices: prevalence, clinical characteristics, and outcomes. Circulation (2005) 112(9 Suppl):I32–6. 10.1161/CIRCULATIONAHA.104.524124 16159839

[29] Yacoub MH A novel strategy to maximize the efficacy of left ventricular assist devices as a bridge to recovery. Eur Heart J (2001) 22(7):534–40 10.1053/euhj.2001.2613 11259141

[30] Birks EJ Tansley PD Hardy J George RS Bowles CT Burke M Left ventricular assist device and drug therapy for the reversal of heart failure. N Engl J Med (2006) 355(18):1873–84. 10.1056/NEJMoa053063 17079761

[31] Birks EJ George RS Firouzi A Wright G Bahrami T Yacoub MH Long-term outcomes of patients bridged to recovery versus patients bridged to transplantation. J Thorac Cardiovasc Surg (2012) 144(1):190–6. 10.1016/j.jtcvs.2012.03.021 22498081

[32] Simon MA Primack BA Teuteberg J Kormos RL Bermudez C Toyoda Y Left ventricular remodeling and myocardial recovery on mechanical circulatory support. J Card Fail (2010) 16(2):99–105. 10.1016/j.cardfail.2009.10.018 20142020 PMC2819986

[33] Valentova M von Haehling S. An overview of recent developments in the treatment of heart failure: update from the ESC Congress 2013. Expert Opin Investig Drugs (2014) 23(4):573–8. 10.1517/13543784.2014.881799 24490905

[34] Dimmeler S Zeiher AM Schneider MD. Review series unchain my heart: the scientific foundations of cardiac repair. J Clin Invest (2005) 115(3):572–83. 10.1172/JCI200524283.572 15765139 PMC1052009

[35] Segers VFM Lee RT Stem-cell therapy for cardiac disease. Nature (2008) 451(7181):937–42 10.1038/nature06800 18288183

[36] Menasché P Hagège AA Vilquin J-T Desnos M Abergel E Pouzet B Autologous skeletal myoblast transplantation for severe postinfarction left ventricular dysfunction. J Am Coll Cardiol (2003) 41(7):1078–83. 10.1016/S0735-1097(03)00092-5 12679204

[37] Dimmeler S Burchfield J Zeiher AM. Cell-based therapy of myocardial infarction. Arterioscler Thromb Vasc Biol (2008) 28(2):208–16. 10.1161/ATVBAHA.107.155317 17951319

[38] Menasché P. Cardiac cell therapy: lessons from clinical trials. J Mol Cell Cardiol (2011) 50(2):258–65. 10.1016/j.yjmcc.2010.06.010 20600097

[39] Pagani FD DerSimonian H Zawadzka A Wetzel K Edge ASB Jacoby DB Autologous skeletal myoblasts transplanted to ischemia-damaged myocardium in humans. Histological analysis of cell survival and differentiation. J Am Coll Cardiol (2003) 41(5):879–88. 10.1016/S0735-1097(03)00081-0 12628737

[40] Dib N Michler RE Pagani FD Wright S Kereiakes DJ Lengerich R Safety and feasibility of autologous myoblast transplantation in patients with ischemic cardiomyopathy: four-year follow-up. Circulation (2005) 112(12):1748–55. 10.1161/CIRCULATIONAHA.105.547810 16172284

[41] Strauer B-E Yousef M Schannwell CM. The acute and long-term effects of intracoronary stem cell transplantation in 191 patients with chronic heart failure: the STAR-heart study. Eur J Heart Fail (2010) 12(7):721–9. 10.1093/eurjhf/hfq095 20576835

[42] Van der Spoel TI Jansen Of Lorkeers SJ Agostoni P van Belle E Gyongyosi M Sluijter JP Human relevance of pre-clinical studies in stem cell therapy; systematic review and meta-analysis of large animal models of ischemic heart disease. Cardiovasc Res (2011) 91(4):649–58. 10.1093/cvr/cvr113 21498423

[43] Koudstaal S Stamceltherapie voor ischemische hartziekten. Cordiaal (2013) 2:40–4.

[44] Jeevanantham V Butler M Saad A Abdel-Latif A Zuba-Surma EK Dawn B Adult bone marrow cell therapy improves survival and induces long-term improvement in cardiac parameters: a systematic review and meta-analysis. Circulation (2012) 126(5):551–68 10.1161/CIRCULATIONAHA.111.086074 22730444 PMC4282649

[45] Van Ramshorst J Bax JJ Beeres SL Dibbets-Schneider P Roes SD Stokkel MP Intramyocardial bone marrow cell injection for chronic myocardial ischemia. JAMA (2009) 301(19):1997–2004 10.1001/jama.2009.685 19454638

[46] Tongers J Losordo DW Landmesser U. Stem and progenitor cell-based therapy in ischaemic heart disease: promise, uncertainties, and challenges. Eur Heart J (2011) 32(10):1197–206. 10.1093/eurheartj/ehr018 21362705 PMC3094549

[47] Radisic M Christman KL. Materials science and tissue engineering: repairing the heart. Mayo Clin Proc (2013) 88(8):884–98. 10.1016/j.mayocp.2013.05.003 23910415 PMC3786696

[48] Kurazumi H Kubo M Ohshima M Yamamoto Y Takemoto Y Suzuki R The effects of mechanical stress on the growth, differentiation, and paracrine factor production of cardiac stem cells. PLoS One (2011) 6(12):e28890. 10.1371/journal.pone.0028890 22216136 PMC3247223

[49] Bergmann O Bhardwaj RD Bernard S Zdunek S Barnabé-Heider F Walsh S Evidence for cardiomyocyte renewal in humans. Science (2009) 324(5923):98–102 10.1126/science.1164680 19342590 PMC2991140

[50] Smits AM van Vliet P Metz CH Korfage T Sluijter JP Doevendans PA Human cardiomyocyte progenitor cells differentiate into functional mature cardiomyocytes: an *in vitro* model for studying human cardiac physiology and pathophysiology. Nat Protoc (2009) 4(2):232–43. 10.1038/nprot.2008.229 19197267

[51] Senyo S Wang M Wu T Lechene CP. Mammalian heart renewal by preexisting cardiomyocytes. Nature (2013) 493(7432):433–6. 10.1038/nature11682.Mammalian 23222518 PMC3548046

[52] Bolli R Chugh AR D’Amario D Loughran JH Stoddard MF Ikram S Cardiac stem cells in patients with ischaemic cardiomyopathy (SCIPIO): initial results of a randomised phase 1 trial. Lancet (2011) 378(9806):1847–57. 10.1016/S0140-6736(11)61590-0 22088800 PMC3614010

[53] Malliaras K Makkar RR Smith RR Cheng K Wu E Bonow RO Intracoronary cardiosphere-derived cells after myocardial infarction: evidence of therapeutic regeneration in the final 1-year results of the CADUCEUS trial (CArdiosphere-Derived aUtologous stem CElls to reverse ventricUlar dySfunction). J Am Coll Cardiol (2014) 63(2):110–22. 10.1016/j.jacc.2013.08.724 24036024 PMC3947063

[54] Gho JMIH Kummeling GJM Koudstaal S Jansen Of Lorkeers SJ Doevendans PA Asselbergs FW Cell therapy, a novel remedy for dilated cardiomyopathy? A systematic review. J Card Fail (2013) 19(7):494–502. 10.1016/j.cardfail.2013.05.006 23834925

[55] Bellin M Marchetto MC Gage FH Mummery CL. Induced pluripotent stem cells: the new patient? Nat Rev Mol Cell Biol (2012) 13(11):713–26. 10.1038/nrm3448 23034453

[56] Janssens S Stem cells in the treatment of heart disease. Annu Rev Med (2010) 61:287–300 10.1146/annurev.med.051508.215152 20059339

[57] McElhinney DB Colan SD Moran AM Wypij D Lin M Majzoub JA Recombinant human growth hormone treatment for dilated cardiomyopathy in children. Pediatrics (2004) 114(4):e452–8. 10.1542/peds.2004-0072 15466071

[58] Castellano G Affuso F Di Conza P Fazio S The GH/IGF-1 axis and heart failure. Curr Cardiol Rev (2009) 5(3):203–15 10.2174/157340309788970306 20676279 PMC2822143

[59] Urbanek K Rota M Cascapera S Bearzi C Nascimbene A De Angelis A Cardiac stem cells possess growth factor-receptor systems that after activation regenerate the infarcted myocardium, improving ventricular function and long-term survival. Circ Res (2005) 97(7):663–73. 10.1161/01.RES.0000183733.53101.11 16141414

[60] Ellison GM Torella D Dellegrottaglie S Perez-Martinez C Perez de Prado A Vicinanza C Endogenous cardiac stem cell activation by insulin-like growth factor-1/hepatocyte growth factor intracoronary injection fosters survival and regeneration of the infarcted pig heart. J Am Coll Cardiol (2011) 58(9):977–86. 10.1016/j.jacc.2011.05.013 21723061

[61] Koudstaal S Bastings MMC Feyen DA Waring CD van Slochteren FJ Dankers PYW Sustained delivery of insulin-like growth factor-1/hepatocyte growth factor stimulates endogenous cardiac repair in the chronic infarcted pig heart. J Cardiovasc Transl Res (2014) 7(2):232–41. 10.1007/s12265-013-9518-4 24395494 PMC3935103

[62] Cittadini A Grossman JD Napoli R Katz SE Strömer H Smith RJ Growth hormone attenuates early left ventricular remodeling and improves cardiac function in rats with large myocardial infarction. J Am Coll Cardiol (1997) 29(5):1109–16. 10.1016/S0735-1097(97)00010-7 9120168

[63] Giusti II Rodrigues CG Salles FB Sant’Anna RT Eibel B Han SW High doses of vascular endothelial growth factor 165 safely, but transiently, improve myocardial perfusion in no-option ischemic disease. Hum Gene Ther Methods (2013) 24(5):298–306. 10.1089/hgtb.2012.221 23944648

[64] Fazio S Sabatini S Capaldo B Vigorito C Giordano A Guida R A preliminary study of growth hormone in the treatment of dilated cardiomyopathy. N Engl J Med (1996) 334(13):809–14. 10.1056/NEJM199603283341301 8596546

[65] Isgaard J Bergh CH Caidahl K Lomsky M Hjalmarson A Bengtsson B. A placebo-controlled study of growth hormone in patients with congestive heart failure. Eur Heart J (1998) 19(11):1704–11. 10.1053/euhj.1998.1123 9857924

[66] Acevedo M Corbalán R Chamorro G Jalil J Nazzal C Campusano C Administration of growth hormone to patients with advanced cardiac heart failure: effects upon left ventricular function, exercise capacity, and neurohormonal status. Int J Cardiol (2003) 87:185–91. 10.1016/S0167-5273(02)00249-8 12559539

[67] Spallarossa P Rossettin P Minuto F Caruso D Cordera R Battistini M Evaluation of growth hormone administration in patients with chronic heart failure secondary to coronary artery disease. Am J Cardiol (1999) 84(4):430–3. 10.1016/S0002-9149(99)00328-8 10468082

[68] Smit JW Janssen YJ Lamb HJ van der Wall EE Stokkel MP Viergever E Six months of recombinant human GH therapy in patients with ischemic cardiac failure does not influence left ventricular function and mass. J Clin Endocrinol Metab (2001) 86(10):4638–43. 10.1210/jcem.86.10.7832 11600518

[69] Kang H-J Kim H-S Zhang S-Y Park K-W Cho H-J Koo B-K Effects of intracoronary infusion of peripheral blood stem-cells mobilised with granulocyte-colony stimulating factor on left ventricular systolic function and restenosis after coronary stenting in myocardial infarction: the MAGIC cell randomised clinical. Lancet (2004) 363(9411):751–6. 10.1016/S0140-6736(04)15689-4 15016484

[70] Tilemann L Ishikawa K Weber T Hajjar RJ. Gene therapy for heart failure. Circ Res (2012) 110(5):777–93. 10.1161/CIRCRESAHA.111.252981 22383712 PMC3594844

[71] Jessup M Greenberg B Mancini D Cappola T Pauly DF Jaski B Calcium upregulation by percutaneous administration of gene therapy in cardiac disease (CUPID): a phase 2 trial of intracoronary gene therapy of sarcoplasmic reticulum Ca2 + -ATPase in patients with advanced heart failure. Circulation (2011) 124(3):304–13. 10.1161/CIRCULATIONAHA.111.022889 21709064 PMC5843948

[72] Greenberg B Yaroshinsky A Zsebo KM Butler J Felker GM Voors AA Design of a phase 2b trial of intracoronary administration of AAV1/SERCA2a in patients with advanced heart failure: the CUPID 2 trial (calcium up-regulation by percutaneous administration of gene therapy in cardiac disease phase 2b). JACC Heart Fail (2014) 2(1):84–92. 10.1016/j.jchf.2013.09.008 24622121

[73] Van Rooij E Olson EN. MicroRNA therapeutics for cardiovascular disease: opportunities and obstacles. Nat Rev Drug Discov (2012) 11(11):860–72. 10.1038/nrd3864 23080337 PMC6813819

[74] Hinkel R Penzkofer D Zühlke S Fischer A Husada W Xu Q-F Inhibition of microRNA-92a protects against ischemia/reperfusion injury in a large-animal model. Circulation (2013) 128(10):1066–75. 10.1161/CIRCULATIONAHA.113.001904 23897866

[75] Van Rooij E Sutherland LB Liu N Williams AH McAnally J Gerard RD A signature pattern of stress-responsive microRNAs that can evoke cardiac hypertrophy and heart failure. Proc Natl Acad Sci U S A (2006) 103(48):18255–60. 10.1073/pnas.0608791103 17108080 PMC1838739

[76] Bonauer A Carmona G Iwasaki M Mione M Koyanagi M Fischer A MicroRNA-92a controls angiogenesis and functional recovery of ischemic tissues in mice. Science (2009) 324(5935):1710–3. 10.1126/science.1174381 19460962

[77] Thum T Gross C Fiedler J Fischer T Kissler S Bussen M MicroRNA-21 contributes to myocardial disease by stimulating MAP kinase signalling in fibroblasts. Nature (2008) 456(7224):980–4. 10.1038/nature07511 19043405

[78] Vrijsen KR Sluijter JPG Schuchardt MWL van Balkom BWM Noort WA Chamuleau SAJ Cardiomyocyte progenitor cell-derived exosomes stimulate migration of endothelial cells. J Cell Mol Med (2010) 14(5):1064–70. 10.1111/j.1582-4934.2010.01081.x 20465578 PMC3822742

[79] Sahoo S Losordo DW. Exosomes and cardiac repair after myocardial infarction. Circ Res (2014) 114(2):333–44. 10.1161/CIRCRESAHA.114.300639 24436429

[80] Harding C Heuser J Stahl P Receptor-mediated endocytosis of transferrin and recycling of the transferrin receptor in rat reticulocytes. J Cell Biol (1983) 97(2):329–39 10.1083/jcb.97.2.329 6309857 PMC2112509

[81] Sluijter JPG Verhage V Deddens JC van den Akker F Doevendans PA. Microvesicles and exosomes for intracardiac communication. Cardiovasc Res (2014) 102(2):302–11. 10.1093/cvr/cvu022 24488559

[82] Lai RC Arslan F Lee MM Sze NSK Choo A Chen TS Exosome secreted by MSC reduces myocardial ischemia/reperfusion injury. Stem Cell Res (2010) 4(3):214–22. 10.1016/j.scr.2009.12.003 20138817

[83] Johnson TD Christman KL. Injectable hydrogel therapies and their delivery strategies for treating myocardial infarction. Expert Opin Drug Deliv (2013) 10(1):59–72. 10.1517/17425247.2013.739156 23140533

[84] Dai W Kay GL Kloner RA The therapeutic effect of cell transplantation versus non-cellular biomaterial implantation on cardiac structure and function following myocardial infarction. J Cardiovasc Pharmacol Ther (2014) 19(4):350–7 10.1177/1074248413517746 24414282

[85] Lam MT Wu JC Biomaterial application in cardiovascular tissue repair and regeneration. Expert Rev Cardiovasc Ther (2013) 10(8):1039–49 10.1586/erc.12.99 23030293 PMC3556462

[86] Christman KL Vardanian AJ Fang Q Sievers RE Fok HH Lee RJ. Injectable fibrin scaffold improves cell transplant survival, reduces infarct expansion, and induces neovasculature formation in ischemic myocardium. J Am Coll Cardiol (2004) 44(3):654–60. 10.1016/j.jacc.2004.04.040 15358036

[87] Bastings MMC Koudstaal S Kieltyka RE Nakano Y Pape ACH Feyen DAM A fast pH-switchable and self-healing supramolecular hydrogel carrier for guided, local catheter injection in the infarcted myocardium. Adv Healthc Mater (2014) 3(1):70–8. 10.1002/adhm.201300076 23788397

[88] Frazier OH Benedict CR Radovancevic B Bick RJ Capek P Springer WE Improved left ventricular function after chronic left ventricular unloading. Ann Thorac Surg (1996) 62(3):675–82. 10.1016/S0003-4975(96)00437-7 8783992

[89] De Jonge N van Wichen DF Schipper MEI Lahpor JR Gmelig-Meyling FHJ Robles de Medina EO Left ventricular assist device in end-stage heart failure: persistence of structural myocyte damage after unloading. An immunohistochemical analysis of the contractile myofilaments. J Am Coll Cardiol (2002) 39(6):963–9. 10.1016/S0735-1097(02)01713-8 11897437

[90] Terracciano CMN Hardy J Birks EJ Khaghani A Banner NR Yacoub MH. Clinical recovery from end-stage heart failure using left-ventricular assist device and pharmacological therapy correlates with increased sarcoplasmic reticulum calcium content but not with regression of cellular hypertrophy. Circulation (2004) 109(19):2263–5. 10.1161/01.CIR.0000129233.51320.92 15136495

[91] Birks EJ Hall JL Barton PJR Grindle S Latif N Hardy JP Gene profiling changes in cytoskeletal proteins during clinical recovery after left ventricular-assist device support. Circulation (2005) 112(9 Suppl):I57–64. 10.1161/CIRCULATIONAHA.104.526137 16159866

[92] Ibrahim M Rao C Athanasiou T Yacoub MH Terracciano CM. Mechanical unloading and cell therapy have a synergistic role in the recovery and regeneration of the failing heart. Eur J Cardiothorac Surg (2012) 42(2):312–8. 10.1093/ejcts/ezs067 22378852

[93] Schipper MEI van Kuik J de Jonge N Dullens HFJ de Weger RA. Changes in regulatory microRNA expression in myocardium of heart failure patients on left ventricular assist device support. J Heart Lung Transplant (2008) 27(12):1282–5. 10.1016/j.healun.2008.09.005 19059107

[94] Lok SI van Mil A Bovenschen N van der Weide P van Kuik J van Wichen D Post-transcriptional regulation of α-1-antichymotrypsin by microRNA-137 in chronic heart failure and mechanical support. Circ Heart Fail (2013) 6(4):853–61. 10.1161/CIRCHEARTFAILURE.112.000255 23640964

[95] Suzuki R Li T-S Mikamo A Takahashi M Ohshima M Kubo M The reduction of hemodynamic loading assists self-regeneration of the injured heart by increasing cell proliferation, inhibiting cell apoptosis, and inducing stem-cell recruitment. J Thorac Cardiovasc Surg (2007) 133(4):1051–8. 10.1016/j.jtcvs.2006.12.026 17382652

[96] Dipla K Mattiello JA Jeevanandam V Houser SR Margulies KB. Myocyte recovery after mechanical circulatory support in humans with end-stage heart failure. Circulation (1998) 97(23):2316–22. 10.1161/01.CIR.97.23.2316 9639375

[97] Pokushalov E Romanov A Corbucci G Prohorova D Chernyavsky A Larionov P Cardiac resynchronization therapy and bone marrow cell transplantation in patients with ischemic heart failure and electromechanical dyssynchrony: a randomized pilot study. J Cardiovasc Transl Res (2011) 4(6):767–78. 10.1007/s12265-011-9283-1 21547598

[98] Monreal G Sherwood LC Sobieski MA Giridharan GA Slaughter MS Koenig SC. Large animal models for left ventricular assist device research and development. ASAIO J (2014) 60(1):2–8. 10.1097/MAT.0000000000000005 24270232

[99] Goldstein AH Monreal G Kambara A Spiwak AJ Schlossberg ML Abrishamchian AR Partial support with a centrifugal left ventricular assist device reduces myocardial oxygen consumption in chronic, ischemic heart failure. J Card Fail (2005) 11(2):142–51. 10.1016/j.cardfail.2004.07.005 15732036

[100] Monreal G Gerhardt MA. Left ventricular assist device support induces acute changes in myocardial electrolytes in heart failure. ASAIO J (2007) 53(2):152–8. 10.1097/MAT.0b013e3180302a8b 17413553

[101] Ghodsizad A Kar BJ Layolka P Okur A Gonzales J Bara C Less invasive off-pump implantation of axial flow pumps in chronic ischemic heart failure: survival effects. J Heart Lung Transplant (2011) 30(7):834–7. 10.1016/j.healun.2011.03.012 21530315

[102] Bartoli CR Giridharan GA Litwak KN Sobieski M Prabhu SD Slaughter MS Hemodynamic responses to continuous versus pulsatile mechanical unloading of the failing left ventricle. ASAIO J (2010) 56(5):410–6 10.1097/MAT.0b013e3181e7bf3c 20613490

[103] Geens JH Jacobs S Claus P Trenson S Leunens V Vantichelen I Partial mechanical circulatory support in an ovine model of post-infarction remodeling. J Heart Lung Transplant (2013) 32(8):815–22. 10.1016/j.healun.2013.05.019 23856219

[104] Tuzun E Bick R Kadipasaoglu C Conger JL Poindexter BJ Gregoric ID Modification of a volume-overload heart failure model to track myocardial remodeling and device-related reverse remodeling. ISRN Cardiol (2011) 2011:831062. 10.5402/2011/831062 22347659 PMC3262518

[105] Sawa Y Miyagawa S Sakaguchi T Fujita T Matsuyama A Saito A Tissue engineered myoblast sheets improved cardiac function sufficiently to discontinue LVAS in a patient with DCM: report of a case. Surg Today (2012) 42(2):181–4. 10.1007/s00595-011-0106-4 22200756

[106] Gojo S Kyo S Nishimura S Komiyama N Kawai N Bessho M Cardiac resurrection after bone-marrow-derived mononuclear cell transplantation during left ventricular assist device support. Ann Thorac Surg (2007) 83(2):661–2. 10.1016/j.athoracsur.2006.06.074 17258005

[107] Miyagawa S Matsumiya G Funatsu T Yoshitatsu M Sekiya N Fukui S Combined autologous cellular cardiomyoplasty using skeletal myoblasts and bone marrow cells for human ischemic cardiomyopathy with left ventricular assist system implantation: report of a case. Surg Today (2009) 39(2):133–6. 10.1007/s00595-008-3803-x 19198991

[108] Anastasiadis K Antonitsis P Argiriadou H Koliakos G Doumas A Khayat A Hybrid approach of ventricular assist device and autologous bone marrow stem cells implantation in end-stage ischemic heart failure enhances myocardial reperfusion. J Transl Med (2011) 9(1):12. 10.1186/1479-5876-9-12 21247486 PMC3034699

[109] Nasseri B Kukucka M Dandel M Knosalla C Potapov E Lehmkuhl H Intramyocardial delivery of bone marrow mononuclear cells and mechanical assist device implantation in patients with end-stage cardiomyopathy. Cell Transplant (2007) 16(9):941–9. 10.3727/096368907783338235 18293893

[110] Fujita T Sakaguchi T Miyagawa S Saito A Sekiya N Izutani H Clinical impact of combined transplantation of autologous skeletal myoblasts and bone marrow mononuclear cells in patients with severely deteriorated ischemic cardiomyopathy. Surg Today (2011) 41(8):1029–36. 10.1007/s00595-010-4526-3 21773889

[111] Ascheim DD Gelijns AC Goldstein D Moye LA Smedira N Lee S Mesenchymal precursor cells as adjunctive therapy in recipients of contemporary LVADs. Circulation (2014) 129:2287–96. 10.1161/CIRCULATIONAHA.113.007412 24682346 PMC4243683

